# Relationship Between Hardness and Impact Strength of Epoxy–Glass Composites Modified with Carbonisate from MDF Pyrolysis

**DOI:** 10.3390/ma19010042

**Published:** 2025-12-22

**Authors:** Agata Wieczorska, Sebastian Drewing

**Affiliations:** Faculty of Marine Engineering, Gdynia Maritime University, Morska St. 81-87, 81-225 Gdynia, Poland; s.drewing@wm.umg.edu.pl

**Keywords:** epoxy–glass composites, MDF carbonisate, Barcol hardness, impact strength, statistical analysis, Kruskal–Wallis test

## Abstract

The study analysed epoxy–glass laminates containing carbonisate produced during medium-density fibreboard (MDF) waste pyrolysis were evaluated with respect to their hardness and their ability to withstand impact loads. All composite samples were prepared manually using a hand-laying method, using two resin–reinforcement ratios (60/40 and 65/35) and carbonisate additives in amounts of 5% and 7.5% by weight (with particle sizes < 500 µm). The mechanical properties were assessed on the basis of hardness tests using the Barcol method and impact tests using the Charpy method. To analyse the results, a normality assessment (Shapiro–Wilk) was performed, followed by a non-parametric analysis of variance based on ranks (Kruskal–Wallis). It was found that an increase in carbonisate content increases the surface hardness of composites while reducing their impact resistance, which confirms the existence of a typical trade-off between stiffness and energy absorption capacity. The most favourable mechanical properties were obtained for a composite containing 7.5% carbonisate material and a resin–reinforcement ratio of 60/40, which was characterised by the highest hardness (35.19 HBa), a moderate impact strength (43.56 kJ/m^2^) and the lowest variability of results. The statistical analysis confirmed significant differences between the tested samples and a quantitative relationship between hardness and impact strength. The results of the study indicate that carbonisate (MDF) using waste material as a filler provides a sustainable means of improving the stiffness and consistency of epoxy–glass composites, with only a negligible effect on their ability to resist fracture.

## 1. Introduction

Polymer composites belong to a group of engineering materials with numerous possible applications, characterised by the possibility of shaping their mechanical, thermal and structural properties by selecting the type of matrix, reinforcement and fillers. Numerous studies published in journals have shown that even a small addition of filler—mineral, natural or carbon—can significantly affect the hardness, impact resistance and general quality durability of the material [1,2,3,4]. The mechanical properties of composites depend primarily on the type and content of the filler and on the quality of the interfacial bond between the matrix and the filler particles [5]. In summary, the cited works indicate that the chemical type, structure and morphology of the filler have a key impact on the balance between hardness and impact strength of polymer composites. Optimising the proportions and uniformity of dispersion of natural, functional and hybrid additives remain one of the main areas of development in the design of modern composite materials with defined mechanical properties.

In epoxy-basalt composites, the introduction of natural and mineral additives at 2.5–10% by weight resulted in a noticeable improvement in impact resistance and impact strength [1]. Similar effects were obtained in epoxy composites modified with nanofillers (e.g., Al_2_O_3_, SiC, magnetic particles), where an increase in surface hardness and stiffness was accompanied by only a slight decrease in impact strength, remaining comparable to that of the unmodified resin [6,7]. The effect of cork addition on the mechanical properties of the composite was also examined [8]. The results showed a gradual decrease in mechanical strength with increasing cork content; however, acceptable performance was maintained for lower filler concentrations, indicating potential for practical applications.

On the other hand, excessive additive concentration or poor adhesion at the phase boundary caused particle agglomeration and local stress concentrations, leading to a decrease in impact strength [9,10]. Previous research on epoxy-based polymer composites has shown that their mechanical properties can be significantly enhanced through the incorporation of suitable fillers, reinforcements and functional additives. For example, the addition of mineral or ceramic nanoparticles at 3–10 wt% can increase hardness and elastic modulus by approximately 15–40%, whereas incorporating short glass fibres at 10–30 wt% typically improves tensile strength by 30–50%. Carbon-based fillers used at 5–15 wt% have been reported to enhance hardness by 15–35% and stiffness by 10–25%. These ranges illustrate the magnitude of modification achievable in epoxy matrices through tailored filler selection.

However, with too high a filler content, the composites became more brittle, which was attributed to matrix discontinuity and deterioration of particle wettability [11,12,13]. In thermosetting polymer matrices such as epoxy resins, the use of hybrid fibre reinforcement in combination with particulate fillers, typically added at 2–10 wt%, allows for a favourable compromise between surface hardness and impact resistance. Studies on composite laminates have shown that the appropriate arrangement of layers and fibre orientation significantly affects the ability to absorb energy during impact [13,14,15]. Comparative studies of particle-filled epoxy and thermoplastic polymer matrices indicate that using around 5 wt% of mineral or carbon-based fillers can increase hardness by 25–40% without markedly reducing impact resistance [2,9,12].

In recent years, considerable attention has been directed toward environmentally friendly fillers, including seashell powder, cork, wood-derived materials and microcrystalline cellulose. Such additives are regarded as functional modifiers, as they not only reduce the overall density of the composite but also influence its mechanical and thermal behaviour. Previous studies indicate that they can effectively replace traditional mineral fillers while maintaining satisfactory mechanical performance [3,8,16]. However, their effect on hardness and impact strength is determined by particle size, which influences the efficiency of load transfer; by the degree of dispersion, as well-dispersed fillers create a more continuous stress-transfer network and therefore promote a more uniform distribution of stresses, whereas agglomerated particles act as local stress concentrators; and by chemical compatibility between the components, since favourable interfacial interactions improve adhesion at the matrix–filler boundary, enabling more effective transfer of mechanical loads [17,18].

Prior work also highlights that both filler size and its volume fraction strongly affect composite behaviour. Appropriately selected nanofiller levels (commonly 3–7 wt%) can enhance matrix cross-linking, limit void formation and promote more uniform transfer of stresses throughout the material [4,19]. These mechanisms are directly related to the mechanical response of epoxy materials and are particularly relevant in the context of this study, as hardness and impact strength strongly depend on the quality of load transfer and the presence of stress concentrators. For example, modification of epoxy resin with Al_2_O_3_ nanoparticles (≈1–3 wt%) has been shown to improve mechanical performance. A study reported increases of about 49.1% in tensile strength and 8.8% in hardness compared to unfilled epoxy [6]. Similarly, the addition of carbon particles (1–2 wt%) to thermosetting polymer matrices resulted in enhanced elastic and plastic properties, including increased stiffness and hardness, indicating improved load-bearing capability [20]. Such findings provide an important reference framework for interpreting the behaviour of epoxy–glass composites modified with carbonised MDF filler investigated in this work.

Recent publications emphasise the growing importance of functional and hybrid fillers, typically incorporated at 3–10 wt%, in modifying the mechanical performance of polymer composites. Kula et al. [21] demonstrated that the addition of hydroxyapatite, fluorine, and silver nanoparticles (2–5 wt%) increases surface hardness by approximately 10–25% and improves impact resistance by 15–20%, without compromising biocompatibility or optical stability. Similar effects were observed for polypropylene composites with chemically modified natural fillers, where improved interfacial adhesion enhanced impact strength by 12–30% and hardness by 8–18% [16]. In resin-based composites, fine fillers added at 2–10 wt% significantly improved micro hardness (typically by 10–35%) and wear resistance, primarily due to uniform dispersion and enhanced load transfer [22]. Conversely, PLA composites containing cork fillers (5–20 wt%) showed a reduction in hardness and impact strength by 10–30%, while simultaneously reducing density by 5–15% and increasing energy absorption capability, highlighting a trade-off between weight reduction and mechanical performance [8]. Hybrid HDPE composites containing combinations of mineral and carbon fillers (5–15 wt%) exhibited a synergistic enhancement of properties, with hardness increasing by 15–25%, stiffness by 10–20% and impact resistance by 10–18%, confirming the effectiveness of multiphase filler systems in improving thermoplastic matrices [23].

Quantitative findings from the cited studies confirm the significance of these effects. Epoxy composites modified with nanofillers such as Al_2_O_3_ or SiC typically show an increase in hardness and stiffness, most often in the range of 10–30%, while maintaining impact strength at a level comparable to the unmodified matrix [6,7]. In contrast, bio-based fillers such as cork or wood flour tend to reduce mechanical strength at higher loadings, with decreases commonly reported in the range of 10–35% [3,8]. Hybrid filler systems combining mineral and carbon-based particles demonstrate a synergistic effect, leading to simultaneous improvements in hardness, stiffness and impact resistance, typically by 10–40% depending on filler ratio and dispersion quality [21,23]. These quantitative trends clearly demonstrate the relevance of filler selection and concentration when analysing the mechanical response of epoxy composites.

Wieczorska et al. [24] examined the effect of carbonisate fraction content on the hardness of epoxy composites, demonstrating that the addition of 5–7.5% by weight increases the surface hardness of the material and improves its structural homogeneity. The sample containing 7.5 wt% filler reached a hardness of ≈33.6 HBa, which represents a clear improvement compared with the unfilled reference material. These results confirmed that a moderate content of fine-grained filler promotes the strengthening of the interfacial bond in the epoxy resin matrix, thereby increasing the material’s resistance to local deformation. In turn, Zuk et al. [25] showed that epoxy–glass laminates modified with 5 wt% recycled rubber exhibit a significant enhancement in energy absorption capacity during dynamic loading. In one of the tested sandwich configurations, the maximum force Fₘₐₓ increased from 2983 N (reference laminate) to 3240 N for the rubber-modified laminate, while the corresponding displacement rose from 1.52 mm to 1.85 mm, confirming a measurable improvement in impact energy dissipation. The authors emphasised that the arrangement of layers containing recycled material determines the balance between stiffness and impact strength of the composite. These results indicate the existence of a typical compromise for composite systems—an increase in hardness and stiffness is often associated with a reduction in impact and fracture resistance. In a subsequent publication [26], the tensile properties of epoxy composites with carbonisate additives (5 wt% and 7.5 wt%) were analysed, assessing the dynamics of deformation in real time. It was shown that increasing the carbonisate content—from 5 wt% to 7.5 wt%—led to a noticeable decrease in elongation at break and tensile strength, which the authors explained as related to local discontinuity of the matrix and the formation of micropores at the phase boundary. The method used to describe and analyse deformation changes as a function of time enabled the identification of boundary points at which the material’s behaviour changes from elastic to brittle. This line of research was complemented by Wieczorska et al. [27], who applied the innovative Kolmogorov–Sinai metric entropy technique to characterise the performance parameters of epoxy–glass composites modified with carbonisate (5 wt% and 7.5 wt%). This method made it possible to describe the non-stationary changes occurring in the material structure under the influence of dynamic loads, which allowed the critical points of degradation and change in the mechanical behaviour of the composite to be determined.

In light of the above observations, it is important to determine the quantitative relationship between hardness and impact strength for the epoxy–glass composite modified with carbonisate. The analysis concerns materials containing two levels of filler content (5 wt% and 7.5 wt%) and two particle-size fractions of carbonisate, which allows us to understand how both the amount and morphology of the filler influence the interaction between these properties. Such an approach enables the identification of the composition range in which an optimal compromise between surface resistance and energy absorption capacity is achieved. The use of statistical methods, such as the Shapiro–Wilk and Kruskal–Wallis tests, provides an objective assessment of these relationships and allows us to evaluate the extent to which carbonisate content and fraction size modify the mechanical performance of the epoxy-based composite.

This work is an extension of previous research analysing how carbonisate addition affects composite hardness, adding a new aspect, the assessment of impact strength and a quantitative analysis of the relationship between these two properties. The statistical approach to the results allows for a more complete understanding of the trade-off between stiffness and impact resistance in epoxy materials modified with carbonisate (MDF) pyrolysis. Among such modifiers, carbonisate derived from pyrolysed MDF waste is particularly noteworthy. Previous studies have shown that this material is carbon-rich and low-density, with a highly porous microstructure that provides substantial adsorption capacity [24,26,27]. Owing to these characteristics, carbonisate can therefore be considered a functional filler with the potential to enhance selected mechanical properties of the polymer matrix.

This study seeks to quantify the correlation between hardness and impact performance in epoxy composites incorporating carbonisate obtained through the pyrolysis of MDF waste based on statistical evaluation techniques. These findings are consistent with our earlier studies on tensile and flexural behaviour of carbonisate-modified laminates, where increasing filler content led to reduced elongation at break and enhanced stiffness [26,27].

## 2. Materials and Methods

The composites were fabricated through the hand lay-up method, in which consecutive layers of reinforcement mat were positioned in a mould and saturated with an epoxy–carbonisate resin mixture.

**Figure 1:**
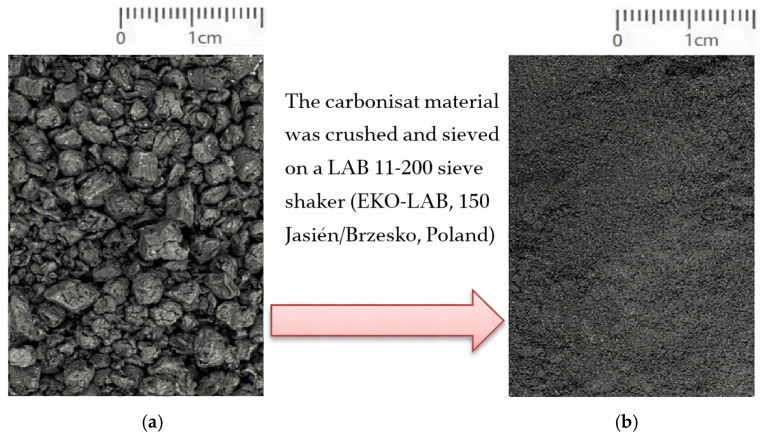
Carbonisate: (**a**) the pyrolysis-derived material in its initial form and (**b**) the material ground and sifted to a particle size of less than 500 µm.

**Figure 2:**
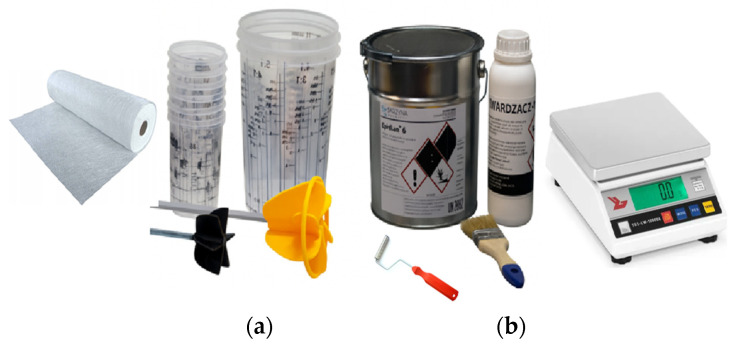
Materials and equipment used in the production of composite materials: (**a**) reinforcement and processing accessories, including glass fibre mat, measuring cylinders, mixers; (**b**) rollers and brushes, epoxy resin system (resin and hardener) and precision laboratory scale.

The research material was prepared as follows:

1. Preparation of carbonisate: a fraction with particle size < 500 µm was obtained by grinding and sieving on a LAB 11-200 sieve shaker (EKO-LAB, Jasień/Brzesko, Poland), Figure 1.
2. Preparation of reinforcement: 10 layers of EM 1002/450/125 emulsion-bound glass mat with a randomly oriented fibre structure were incorporated into the laminate, Figure 2.
3. Preparation of the matrix: Epidian 6 epoxy resin (CIECH Sarzyna S.A., Nowa Sarzyna, Poland) combined with the aliphatic-amine hardener Z-1, added at 13 g per 100 g of resin in accordance with the manufacturer’s recommendations. Figure 2.
4. Preparation of the composite comprising epoxy resin (matrix), reinforcement (mat) and carbonisate material (filler). The carbonisate material was added in portions to the resin, mixing mechanically at low speed (approx. 200 rpm) for several minutes. Additional manual mixing allowed the filler to be dispersed and a homogeneous suspension to be obtained before impregnating the mat.

Figure 2 shows the materials used to produce composites, including glass mat as reinforcement, epoxy resin with a hardener as a matrix, and basic accessories used during the manual lamination process.

The carbonisate material used in the research was a product of the pyrolysis of MDF, obtained under conditions of limited oxygen access. As a result of the pyrolysis process, volatile compounds were removed from the parent material, and the residue took the form of a porous, carbon-rich solid (carbonised material). The obtained carbonisate material had a bulk density of 242 kg/m^3^ [28], a volatile content of 14.6% of the sample weight [29], a total moisture content of 1.6% [30], a calorific value of 29,220 J/g and a combustion heat of 29,900 J/g in working condition. The loss on ignition was 93.87% [31], which indicates a high proportion of organic components and low ash content. These characteristics indicate that the material has a high carbon content, a small mineral fraction and significant porosity, which may contribute to its low density and good adsorption properties. Low moisture content and relatively low volatile matter content contribute to its stability in the epoxy matrix environment.

**Figure 3:**
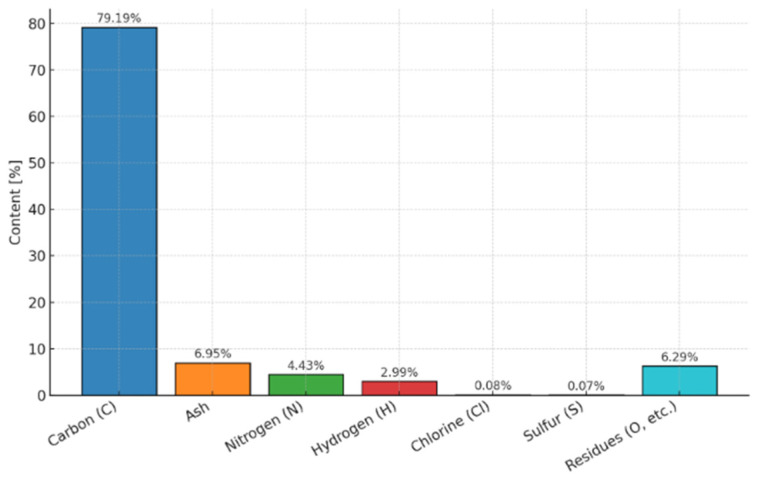
Elemental profile of carbonisate produced through MDF pyrolysis is presented as a bar chart. Carbon is the dominant component (79.19%), followed by ash and minor elements.

The carbonisate obtained through MDF-board pyrolysis exhibits the elemental composition illustrated in Figure 3. The results of the analysis reveal that the material contains a dominant carbon fraction and small amounts of other elements, such as nitrogen, hydrogen and oxygen. The presence of sulphur and chlorine is negligible, which confirms the low level of inorganic impurities.

Two resin–reinforcement ratios (60/40 and 65/35) were used to represent the fibre-dominated and resin-dominated systems, respectively. This selection allows the influence of constant modification (carbonisate < 500 µm with a share of 5 and 7.5%) to be separated from the influence of the matrix content. With the type of mat, number of layers (10) and curing conditions remaining unchanged, the only system variable is the resin–reinforcement ratio, which allows for a clear assessment of the hardness–impact strength trade-off.

All samples, including reference samples (without filler) and samples with 500 µm carbonisate, were cured at room temperature. The resulting composites were used to evaluate the relationship between hardness and impact strength, enabling an evaluation of the influence of carbonisate incorporation on the trade-off between stiffness and the material’s ability to absorb energy.

The composition of the hand-laminated composites, including the percentage share of each component and the sample designations, is listed in Table 1.

**Figure 4:**
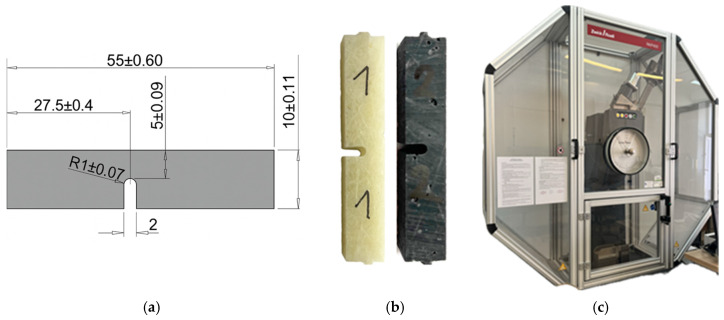
(**a**) Sample geometry and size for impact testing, (**b**) composite test specimens for impact testing, (**c**) Charpy pendulum hammer, model RKP450.

The experimental programme comprised impact testing and hardness evaluation performed using the Barcol method in accordance with GB/T 3854-2005 [32]. To avoid structural alterations caused by heat during specimen preparation, water-jet cutting was employed to produce the test samples. U-notched specimens for impact measurements were fabricated following the PN-EN ISO 179-1:2010E standard [33]. The impact tests were carried out on an RKP450 Charpy pendulum impact tester equipped with TestXpert II software version 3.61, from Zwick and Roell (Figure 4).

## 3. Results

### 3.1. Impact Strength Results

**Figure 5:**
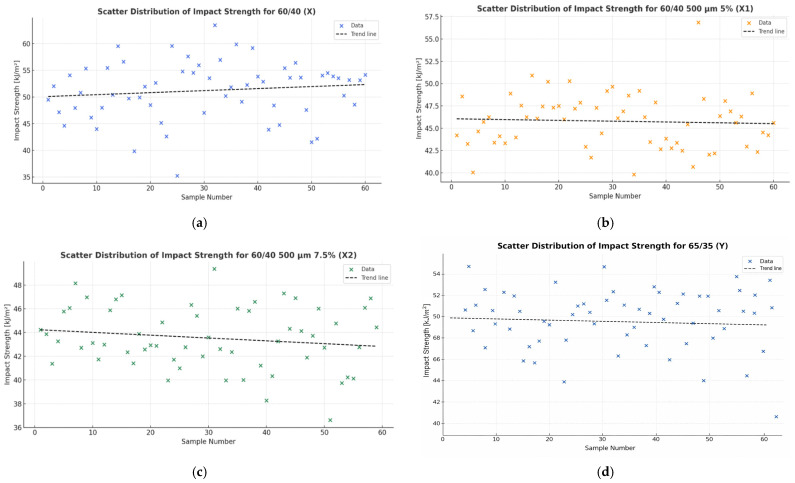

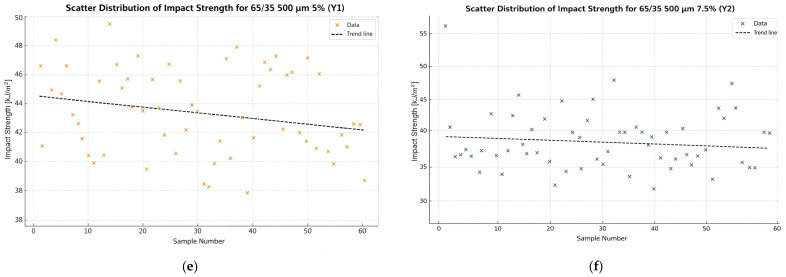
Scatter plots showing the impact strength values [kJ/m^2^] of composite samples for variant 60/40, (**a**) X (reference material without carbonisate), (**b**) X1 (material with 5 wt% carbonisate) and (**c**) X2 (material with 7.5 wt% carbonisate), and for variant 65/35, (**d**) Y (reference material without carbonisate), (**e**) Y1 (material with 5 wt% carbonisate) and (**f**) Y2 (material with 7.5 wt% carbonisate).

Impact strength measurements were performed for two variants of composite materials. For each variant, 60 samples were prepared (labelled as X, X1, X2 and Y, Y1, Y2). Figure 5 depicts the distribution of impact strength results for the tested materials, while Table 2 compiles the average values recorded in these tests.

### 3.2. Barcol Hardness Results

**Figure 6:**
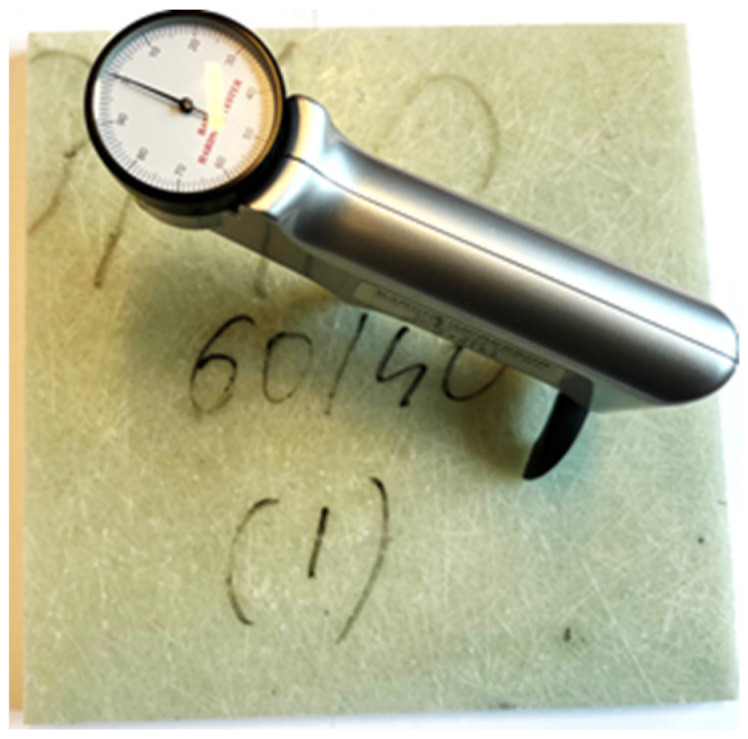
A composite sample measuring 200 × 200 mm prepared for hardness measurement using the Barcol method.

Hardness measurements were carried out using the Barcol technique, as specified in GB/T 3854-2005, which is commonly used in plastics testing for control, design and research purposes. The measurement consisted of pressing the measuring tip (stylus) into the tested surface under spring pressure, and the result was read on a scale ranging from 1 to 100 units. An example sample for hardness measurements with dimensions of 200 × 200 mm is shown in Figure 6.

**Figure 7:**
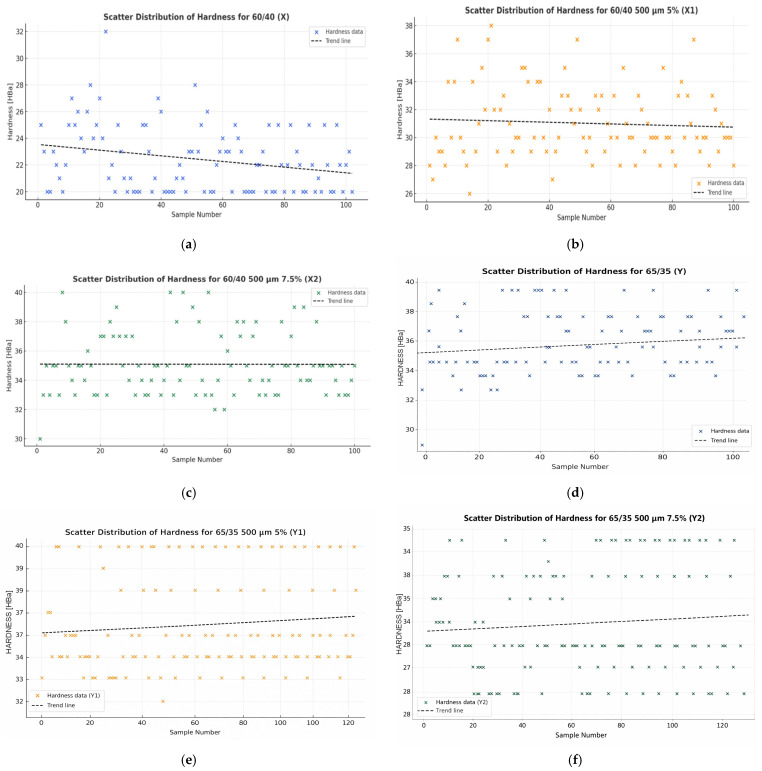
Scatter plots showing the hardness values [HBa] of composite samples for variant 60/40 (**a**) X (material without carbonisate), (**b**) X1 (material formulated with 5 wt% carbonisate), (**c**) X2 (material incorporating 7.5 wt% carbonisate) and of composite samples for variant 65/35 (**d**) Y (material without carbonisate), (**e**) Y1 (material formulated with 5 wt% carbonisate), (**f**) Y2 (material incorporating 7.5 wt% carbonisate).

Hardness measurements were performed for two variants of composite materials. For each variant, 100 measurements were performed, marked as X, X1, X2 and Y, Y1, Y2. Figure 7 show scatter plots illustrating the hardness values of the obtained materials. The average hardness values obtained in the tests are summarised in Table 3.

### 3.3. Determination of the Number of Specimens for Impact Toughness and Hardness Testing

The sample size *n* was calculated for all materials using Stein’s calculation model when the distribution of the population is normal or deviates from normality [34]:

(1) $$n\geq \frac{t_{\alpha ,n-1\;}^{2}S_{\left(x\right)}^{2}}{d^{2}}$$

where t corresponds to the critical value from Student’s t-distribution, α is the adopted significance level, n − 1 indicates the degrees of freedom, s stands for the standard deviation and d specifies the maximum allowable measurement error.

Parametric statistical methods, Student’s *t*-tests and ANOVA were used to determine the sample size for impact testing of material X. The following parameters were obtained: S = 5.43 (for the first 60 initial measurements performed on material X), *d* = 1.375 and *t* = 1.96 for a statistical significance level of α = 95%. On the basis of Equation (1), the calculated amount of material tested was *n* = 59.9. For further analysis, *n* = 60 was adopted.

In order to determine the sample size for measuring the hardness of material X1, parametric statistical tests were used: Student’s *t*-tests and ANOVA. The following values were obtained: S = 2.58 (for the first 100 measurements performed on sample X1), *d* = 0.505 and *t* = 1.96 for a statistical significance level of α = 95%. Based on Formula (1), the calculated amount of material tested was *n* = 100.27. For further analysis, *n* = 100 was adopted.

Table 4 summarises the calculated number of specimens (*n*) required for the impact and hardness tests for each composite configuration.

### 3.4. Evaluation of Distribution Normality for Mechanical Test Results

The Shapiro–Wilk test [35] is widely recognised as one of the most effective tools for assessing the normality of a random variable distribution. Its high statistical power means that, for a given significance level α, it allows the H_0_ hypothesis to be rejected more effectively if it is false. The test results are summarised in Table 5 and Table 6.

For the purposes of this test, α was set to α = 95%.

The hypothesis framework was defined as the following:

**H0.**  *The studied parameter exhibits a normal distribution.*

**H1.**  *The studied parameter does not exhibit a normal distribution.*

**Figure 8:**
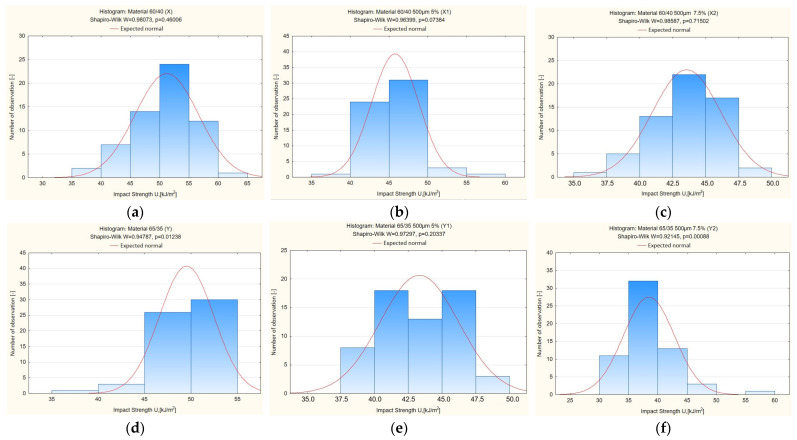
Distribution of impact strength in the examined composite materials, shown as histograms (**a**) material X, (**b**) material X1, (**c**) material X2, (**d**) material Y, (**e**) material Y1, (**f**) material Y2.

Conclusion: Based on the Shapiro–Wilk test results, the following was determined:

- According to the Shapiro–Wilk test results summarised in Table 5 and Figure 8, all *p*-values for materials X, X1, X2 and Y1 exceed the 0.05 threshold, supporting the acceptance of the null hypothesis. Hence, the impact strength variables for these composites exhibit normal distribution characteristics.
- As presented in Table 5 and Figure 8, materials Y and Y2 produced *p*-values lower than 0.05. This necessitates rejecting the null hypothesis and accepting the alternative hypothesis, meaning that the impact strength measurements for these materials deviate from normality.

Conclusion: Because the assumption of normality is not met for all sample groups, parametric tests are inappropriate for comparing the results. Further examination will thus rely on non-parametric procedures [36].

**Figure 9:**
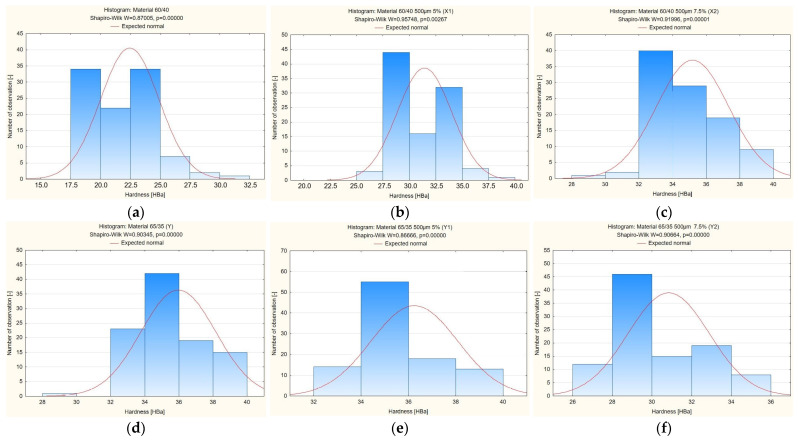
Histograms of the hardness distribution of the tested composite materials (**a**) material X, (**b**) material X1, (**c**) material X2, (**d**) material Y, (**e**) material Y1, (**f**) material Y2.

Figure 9 shows histograms of the impact strength distribution for the tested composite materials. For samples X, X1, X2 and Y1, the shape of the distribution is close to normal, as confirmed by the Shapiro–Wilk test results (*p* > 0.05). The histograms of these samples are characterised by a symmetrical distribution of values around the mean and a small standard deviation.

In contrast, for samples Y and Y2, deviations from the symmetry of the distribution and the presence of extreme values are observed, which is reflected in the results of the normality test (*p* < 0.05). This indicates a lack of normality in the distribution of the impact strength variable in these cases.

The graphical analysis therefore confirms the conclusions of the Shapiro–Wilk test presented in Table 5.

Conclusion: According to the Shapiro–Wilk probabilities presented in Table 6 and Figure 9, each material exhibited a *p*-value under the 0.05 threshold. Thus, the null hypothesis is not supported, and the alternative hypothesis applies—indicating non-normal distribution of hardness values. For this reason, parametric tests are inappropriate, and further analysis will utilise non-parametric approaches [36].

Figure 9 shows histograms of the hardness distribution for the tested composite materials. The results of the Shapiro–Wilk test (Table 6) showed that for all analysed samples, the *p*-values are less than 0.05, which means that the hardness variable distribution is not normal. The histograms of all samples (X, X1, X2, Y, Y1 and Y2) confirm these results—the distributions are asymmetrical, and in some cases, extreme values are visible. This means that the hardness of the tested composites does not have a normal distribution, and therefore, non-parametric tests must be used for further comparative analyses. The graphical analysis thus confirms the conclusions of the Shapiro–Wilk test presented in Table 6.

### 3.5. Analysis of Impact Strength and Hardness Differences Using Non-Parametric Tests

Since some of the distributions did not meet the assumption of ‘normality’ and the variances proved to be heterogeneous, non-parametric tests were used to analyse the impact values: ‘Kruskal–Wallis ANOVA and median test’. A null hypothesis was assumed, according to which the measures of location (distribution) of the tested feature do not differ significantly between groups:

**H0.**  *F1 = F2 = F3 = F4 = F5 = F6, all groups share an identical distribution.*

**H1.**  *F1 ≠ F2 ≠ F3≠ F4 ≠ F5 ≠ F6, at least one distribution is different, meaning the samples do not originate from a single population.*

According to the Kruskal–Wallis ANOVA and the median test results for impact strength (Table 7), the following was found:

- The analyses showed that samples X and Y, X1 and X2, as well as Y1 and X2, do not differ significantly. With *p*-values greater than 0.05, the data do not provide evidence to reject the null hypothesis.
- For the other tested combinations—namely X and X1; X and X2; X and Y1; X and Y2; X1 and X2; X1 and Y1; X1 and Y2; X2 and Y; X2 and Y2; Y and Y1; Y and Y2; and Y1 and Y2—the *p*-values were less than 0.05. This provides grounds to reject the null hypothesis, demonstrating that these samples differ significantly.

**Figure 10:**
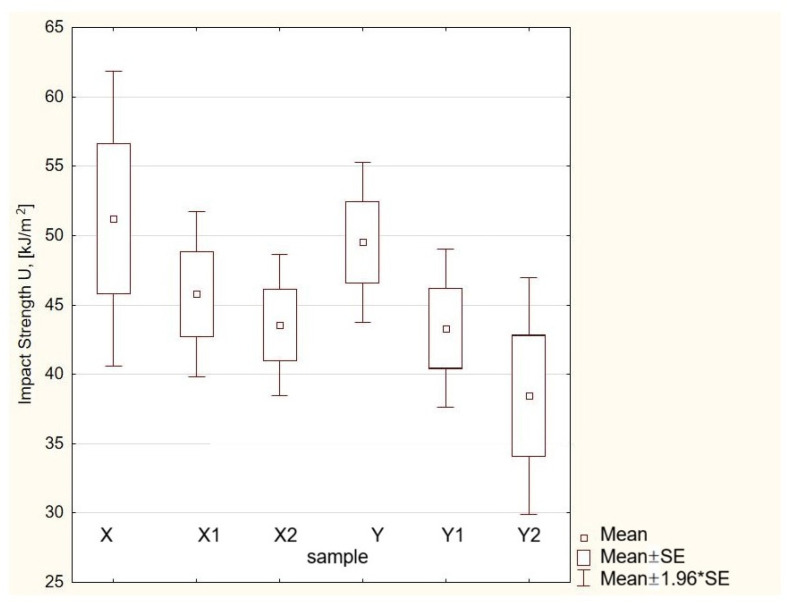
Box-and-whisker representation of impact strength. The term mean ± SE specifies the mean together with its standard error, whereas mean ± 1.96 × SE represents the corresponding confidence bounds.

Figure 10 shows a box-and-whisker plot depicting the distribution of impact strength values for composites X (60/40) and Y (65/35) with 5% and 7.5% carbonisate material (500 μm fraction) added.

Analysis of the Kruskal–Wallis test results (Table 7) and the box-and-whisker plot (Figure 10) showed a statistically significant variation in impact strength (*p* < 0.05) between the tested composites formulated with ratios of X/60/40 and Y/65/35. The addition of 500 μm carbonisate material affected the impact strength distribution—in particular, it was noted that an increase in the carbonisate material content to 7.5% (samples X2 and Y2) resulted in greater variation in the results and a reduction in the mechanical stability of the material. The most stable impact properties were exhibited by sample X2, which was characterised by the smallest dispersion of values (as confirmed by the shape of the box-whisker plot) and no significant differences compared to X1 and Y1. This means that the 60/40 composite with 7.5% carbonisate additive (sample X2) retained the most uniform and repeatable mechanical properties, despite the increased filler content.

Hardness results, summarised in Table 8 and evaluated through the Kruskal–Wallis ANOVA and median test, demonstrate the following:

- The comparisons involving X2 and Y, X2 and Y1, Y1 and Y, as well as X1 and Y2 resulted in *p*-values above the 0.05 threshold. Consequently, the null hypothesis remains valid, meaning that these sample groups do not differ significantly.
- For the other tested combinations—namely, X and X1; X and X2; X and Y; X and Y1; X and Y2; X1 and X2; X1 and Y; X1 and Y1; X2 and Y2; Y and Y2 and Y1 and Y2—the *p*-values were less than 0.05. This provides grounds to reject the null hypothesis, demonstrating that these samples differ significantly.

**Figure 11:**
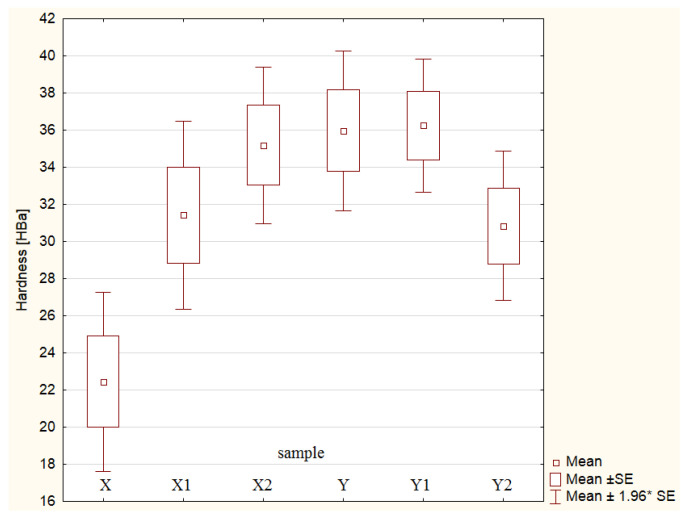
Box-and-whisker representation of hardness values. The expression mean ± SE refers to the mean and its standard error; mean ± 1.96 × SE defines the confidence interval.

Analysis of the Kruskal–Wallis test results (Table 8) and the box-and-whisker plot (Figure 11) showed that there are differences supported by statistical evidence in hardness (*p* < 0.05) between the tested composites with different proportions of components (X/60/40 and Y/65/35). The obtained *p*-values indicate that for most of the compared sample pairs, the differences are statistically significant, which means that both the carbonisate content and the resin–reinforcement ratio have a significant impact on the hardness of the material.

The analysis of the results shows that an increase in the carbonisate content to 7.5% in 60/40 composites (sample X2) causes a noticeable increase in hardness while reducing the spread of results, which indicates better structural homogeneity and good bonding of the filler with the epoxy matrix. However, in the case of composites with a 65/35 composition (Y series), greater variation in hardness values is observed, especially for sample Y2, which may indicate a deterioration in the homogeneity of the material with a higher filler content.

The highest average hardness and the most stable results were obtained for sample X2 (60/40 + 7.5% carbonisate, 500 μm fraction), which was confirmed by both the Kruskal–Wallis test results and the shape of the box-whisker plot (Figure 11). This means that in this configuration, the composite was characterised by the best combination of stiffness and homogeneity, and the addition of carbonisate in the optimal amount improved the surface properties of the material without compromising its structural stability.

In order to provide a more complete description of the impact test results, basic statistical measures were calculated, such as mean value, median, standard deviation, minimum and maximum values, quartiles and coefficients of variation. These parameters are summarised in Table 9 for impact strength and in Table 10 for hardness measurements. These data enable the assessment of the diversity of mechanical properties of the tested materials and the comparison of the homogeneity of individual sample series.

The values of the positional volatility coefficient V_Q_ were calculated using the following formula:

(2) $$V_{Q}=\frac{Q}{M_{e}}\cdot 100\%$$

where V_Q_ is the positional coefficient of variation. It is based on the quotient of the interquartile range and the median. It does not take into account extreme results; positional measures are not sensitive to outliers (extreme values); Q is the interquartile range, and *Me* is the median value of the variable.

(3) $$V_{S}=\frac{\sigma }{\bar{x}}\cdot 100\%$$

where *σ* is the standard deviation; $\bar{x}$ is the arithmetic average of the variable.

Being a percentage-based, dimensionless measure, the coefficient of variation enables the assessment of how diverse different statistical characteristics are. It indicates the extent of data scatter relative to the magnitude of the mean value.

The following interpretation of the Vs coefficient was adopted:

- <26%—low volatility,
- (26–45%)—average volatility,
- (46–100%)—high volatility,
- >100%—very high volatility.

**Figure 12:**
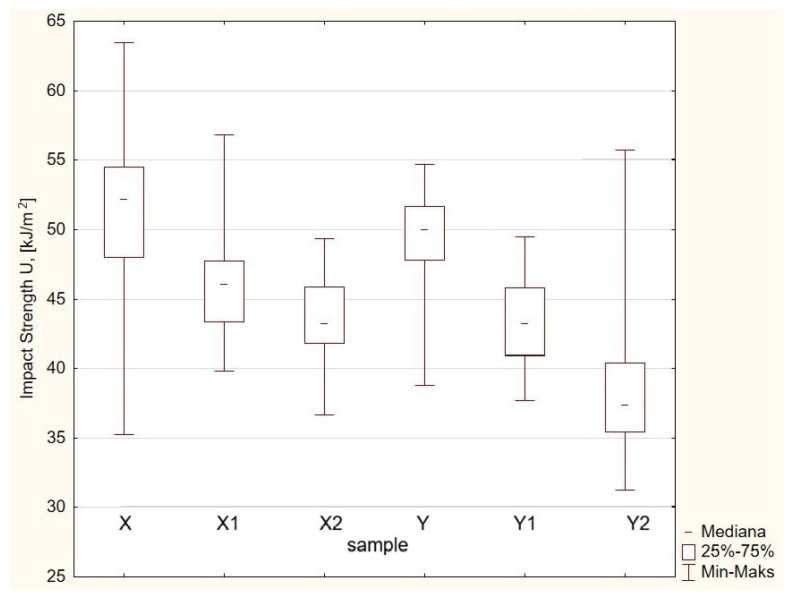
Box-and-whisker plot illustrating the impact strength of the tested materials.

Figure 12 presents a graphical comparison of the impact strength values of the tested composites.

Statistical analysis of impact strength variability measures (Table 9) and box-and-whisker plots (Figure 12) showed clear differences in the impact strength properties of the tested composites depending on their composition and carbonisate content. The highest average impact strength was obtained for sample X (51.23 kJ/m^2^), while the lowest was obtained for sample Y2 (38.46 kJ/m^2^). With an increase in carbonisate content from 0 to 7.5%, a systematic decrease in impact strength was observed in both the X (60/40) and Y (65/35) series. This means that increased filler content limits a composite’s ability to absorb impact energy.

The values of the coefficients of variation (Vs, V_Q_) confirm that samples X2 and Y1 were characterised by the smallest dispersion of results, which indicates greater mechanical homogeneity of these composites. The greatest stability of results was obtained for sample X2 (Vs = 5.97%, V_Q_ = 4.59%), which confirms the high repeatability of impact properties while maintaining a moderate level of fracture energy.

Examination of the data showed that introducing 5–7.5% carbonisate led to a decrease in impact strength, but in the case of the 60/40 system, the most stable and homogeneous distribution of values was obtained. This indicates that, in this range of composition, it is possible to achieve a favourable compromise between hardness and impact resistance.

**Figure 13:**
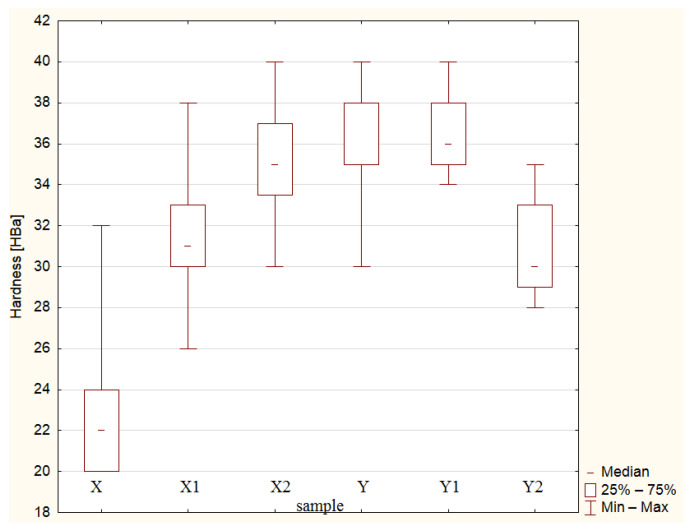
Hardness data of the tested materials represented by box-and-whisker plot.

The analysis of hardness results (Table 10) and box-and-whisker diagrams (Figure 13) confirmed the significant impact of both the resin–reinforcement ratio and the carbonisate content on how the composites behave under mechanical loading. The lowest average hardness was recorded for sample X (22.45 HBa), while the highest was recorded for sample Y1 (36.25 HBa). In series X (60/40), a systematic increase in hardness was observed with increasing carbonisate content—from 22.45 HBa (X) to 35.19 HBa (X2). In contrast, in series Y (65/35), the hardness values reach a plateau, and with a further increase in the carbonisate content (Y2), there is a slight decrease in the average values, which may be due to local structural heterogeneity.

The values of the Vs and V_Q_ coefficients of variation are lowest for samples Y1 and X2, which confirms the good stability and repeatability of the results in these series. The increase in carbonisate in 60/40 composites therefore contributes to increased hardness and improved material cohesion, while in the 65/35 system, too much filler leads to a deterioration in homogeneity and a reduction in load-bearing efficiency in the composite structure.

Based on the analyses conducted, it can be concluded that the most favourable hardness properties were obtained for sample X2 (60/40 + 7.5% carbonisate), which was characterised by a high average hardness value with low variability of results, confirming the effectiveness of this configuration in improving the surface stiffness of the material.

The summary of impact strength and hardness results indicates the existence of a compromise between impact resistance and stiffness typical for composite materials. An increase in carbonisate content leads to increased hardness but, at the same time, reduces impact strength. The most balanced mechanical properties were obtained for sample X2 (60/40 + 7.5% carbonisate), which combines high hardness with good structural stability and moderate loss of impact strength.

### 3.6. Microstructure Analysis (SEM)

Microstructure imaging was performed using a ZEISS EVO MA 15 scanning electron microscope (Gdynia Maritime University). Samples were mounted on stubs using conductive tape and imaged in high vacuum mode at a voltage of 20 kV, an SE detector, and a working distance of approximately 13 mm. Magnification was approximately 50×. Sample orientation was set by the microscope operator.

**Figure 14:**
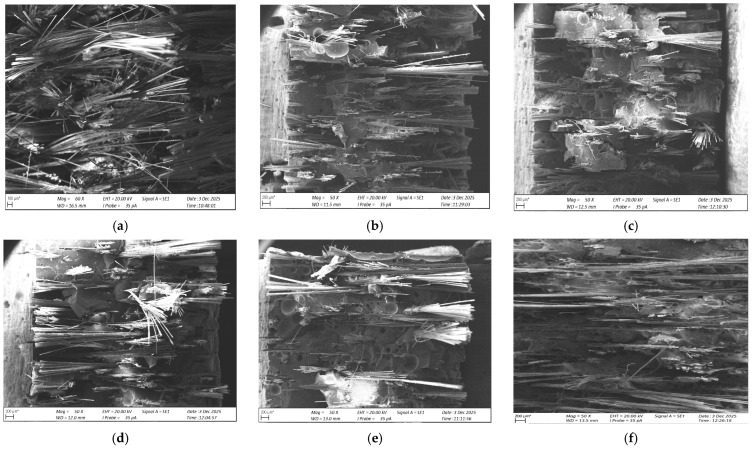
SEM micrographs of fracture surfaces of epoxy–glass composites with different resin–reinforcement ratios and carbonisate contents: (**a**) 60/40—0 wt% carbonisate, reference laminate; (**b**) 60/40—5 wt% carbonisate; (**c**) 60/40—7.5 wt% carbonisate; (**d**) 65/35—0 wt% carbonisate, reference laminate; (**e**) 65/35—5 wt% carbonisate; (**f**) 65/35—7.5 wt% carbonisate. * Scale bar corresponds to the image as displayed (200 µm).

Figure 14 presents the SEM micrographs of the fracture surfaces of epoxy–glass composites for both resin–reinforcement ratios (60/40 and 65/35) and for different carbonisate contents (0%, 5%, and 7.5%). Clear differences in the fracture morphology can be observed as the filler content increases.

The SEM micrographs presented in Figure 14 show the fracture surfaces of the epoxy–glass laminates for both resin–reinforcement ratios (60/40 and 65/35) and carbonisate contents of 0, 5 and 7.5 wt%. Clear and systematic changes in the microstructure can be observed as a function of both the reinforcement ratio and the amount of carbonisate added.

For the 60/40 system, the reference laminate without carbonisate (Figure 14a) displays a fracture surface dominated by long, exposed glass fibres with limited resin coverage. Numerous fibre pull-out areas and local voids indicate incomplete impregnation and a ductile-like fracture with fibre bundles separating along the laminate thickness. Introducing 5 wt% carbonisate (Figure 14b) leads to visible improvements in consolidation: carbonisate particles fill inter-fibre spaces, void content is reduced, and the resin adheres more closely to the fibres. The fracture becomes more compact, with shorter pull-out lengths and more uniform breakage of fibre ends, confirming enhanced wet-out and stiffer local behaviour. At 7.5 wt% carbonisate (Figure 14c), the fracture surface becomes more homogeneous, with continuous matrix–filler–fibre bridges indicating strong interfacial adhesion. Fibre breakage is more uniform and brittle, consistent with the highest hardness and reduced impact strength in this group.

For the 65/35 laminates, the unmodified composite (Figure 14d) shows denser fibre packing due to the higher reinforcement content, accompanied by small regions of incomplete wet-out and voids resulting from the more demanding impregnation during hand lay-up. With the addition of 5 wt% carbonisate (Figure 14e), filler particles are present throughout the fracture surface, although some local agglomerates appear due to the reduced resin volume. Fibre pull-out is further reduced, and the structure becomes more compact, yet still slightly less homogeneous than for the 60/40 system at the same filler level. Increasing the carbonisate content to 7.5 wt% (Figure 14f) results in a dense and cohesive microstructure with well-embedded filler, shortened fibre pull-out, and distinct brittle fracture planes.

Overall, the SEM observations show that increasing the carbonisate content leads to improved fibre–matrix bonding, reduced pull-out, fewer voids, and a more uniform fracture appearance. These changes are accompanied by a transition from more ductile to more brittle fracture behaviour and are fully consistent with the mechanical trends identified in the study. Furthermore, the 60/40 system demonstrates slightly better homogeneity than the 65/35 system, which is attributed to easier resin flow and more efficient wet-out during laminate fabrication.

## 4. Analysis of Results and Relationships Between Hardness and Impact Strength

The mechanical property analysis of carbonisate-modified epoxy–glass composites produced from pyrolysed MDF revealed the influence of filler proportion on both hardness and resistance to impact loading. The results obtained, presented in Table 9 and Table 10 and Figure 13 and Figure 14, indicate clear, systematic changes in these properties depending on the carbonisate content and the resin–reinforcement ratio.

### 4.1. Comparison of Hardness and Impact Strength Values

A comparison of average values showed that increasing the carbonisate content leads to an increase in surface hardness with a simultaneous decrease in impact strength. In the series of composites with a resin–reinforcement ratio of 60/40 (X), a clear increase in hardness was observed as the amount of carbonisate additive increased (from 0 to 7.5%), while impact strength decreased systematically. A similar trend was observed for the 65/35 (Y) series, but the changes in hardness were less pronounced, and the impact strength values showed greater variation. Graphical analysis (Figure 13 and Figure 14) confirmed that as the carbonisate content increased, the distribution of hardness values became more uniform, while the distribution of impact strength became slightly wider. The greatest stability of mechanical properties was obtained for composite X2 (60/40 + 7.5% carbonisate), which was characterised by the smallest spread of hardness results and a moderate decrease in impact strength.

**Figure 15:**
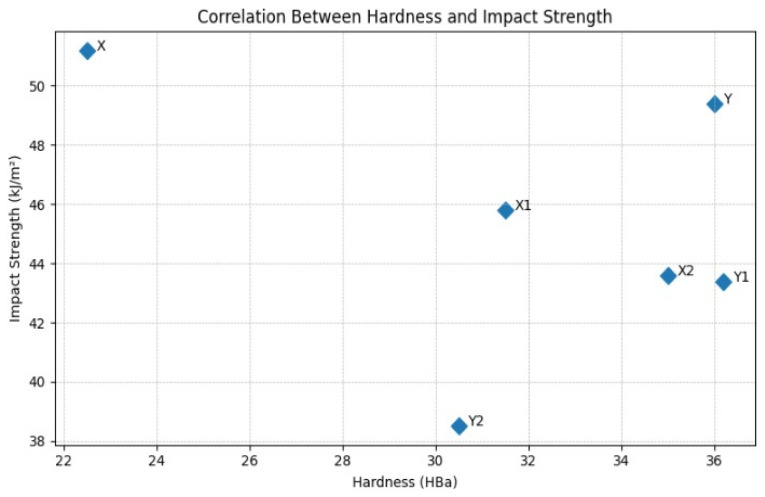
Correlation between Barcol hardness and impact strength for epoxy–glass composites modified with carbonisate systems 60/40 (X, X1, X2) and 65/35 (Y, Y1, Y2); 0, 5, 7.5 wt%.

To make the relationship between Barcol hardness and impact strength explicit rather than implicit, the mean values obtained for all composite variants were plotted in a hardness–impact strength correlation diagram (Figure 15). The plot clearly demonstrates a negative relationship between the two properties: an increase in hardness is accompanied by a systematic decrease in impact strength. The reference composites (0 wt% carbonisate) show the highest impact resistance and the lowest hardness, while composites containing 7.5 wt% exhibit the opposite tendency. This confirms the trade-off between surface stiffness and energy absorption capacity described earlier in the literature and observed in this study.

The results obtained confirm the observations from previous studies [24], which found that a composite with a resin–reinforcement ratio of 60/40 and a 7.5% addition of carbonisate material (<500 µm) exhibits the highest hardness and homogeneity. In the present study, this sample also showed the greatest stability of mechanical properties, which confirms the repeatability of the reinforcing effect of carbonisate.

### 4.2. The Nature of the Relationship Between Hardness and Impact Strength

A comparative analysis of hardness and impact strength results indicates a negative correlation between these properties. As hardness increases due to higher carbonisate content, impact resistance decreases. This phenomenon reflects the typical trade-off between stiffness and energy absorption capacity in composite materials. The more evident effect observed in composites with increased matrix content (60/40) shows that the interplay between the epoxy phase and the filler is a key factor governing their mechanical characteristics.

### 4.3. Assessment of the Significance of Differences

Non-parametric tests (Kruskal–Wallis and median tests) showed that the differences between individual series of materials are statistically significant (*p* < 0.05). The exception was a few pairs of samples in which the *p*-values exceeded the significance level, which means that there were no significant differences between them. The results confirm that both the carbonisate content and the resin–reinforcement ratio have a significant and systematic effect on the hardness and impact strength of the tested composites.

### 4.4. Summary of the Analysis

An examination of the results leads to the following conclusions:

- As the amount of carbonisate rises, the composites exhibit increased surface hardness accompanied by a reduction in impact strength.
- The most favourable mechanical properties were obtained for composite X2 (60/40 + 7.5% carbonisate material), which is characterised by high hardness, good homogeneity and an acceptable level of impact strength.
- The relationship between hardness and impact strength is negative, confirming the existence of a trade-off between stiffness and fracture resistance.
- The use of non-parametric methods in data analysis was fully justified and allowed for a reliable assessment of the significance of differences between the studied groups.

The microstructural observations presented in Section 3.6 support these findings. The SEM analysis revealed reduced fibre pull-out, fewer voids and more compact fracture morphology as the carbonisate content increased, confirming improved matrix–fibre bonding and a transition towards more brittle behaviour. These features directly correspond to the measured increase in hardness and the simultaneous decrease in impact strength.

## 5. Conclusions

The use of carbonate derived from the pyrolysis of MDF boards as a filler in epoxy–glass composites significantly affects their mechanical properties. An increase in carbonisate content from 0 to 7.5% by weight causes a systematic increase in surface hardness and a simultaneous decrease in impact strength, which confirms the existence of a trade-off between stiffness and impact resistance.

Statistical analysis showed that not all data sets are normally distributed; therefore, the use of non-parametric methods (Kruskal–Wallis test and median test) was justified. The test results confirmed the significance of the differences identified among the samples tested, both in terms of hardness and impact strength.

The highest hardness values (35.19 HBa) and the most uniform mechanical properties (lowest Vs and V_Q_ values) were obtained for a composite containing 7.5% carbonisate material with a resin–reinforcement ratio of 60/40. This means that a moderate addition of filler improves the stiffness of the structure without significantly compromising its cohesion.

An increase in carbonisate proportion leads to a decline in impact resistance (from 51.23 kJ/m^2^ to 38.46 kJ/m^2^). This behaviour arises from the diminished stress-relaxation capacity of the epoxy matrix and the resulting growth of the brittle phase fraction.

An analysis of the relationship between hardness and impact strength confirms the existence of a negative relationship between these properties. As the material becomes harder, its ability to dissipate impact energy diminishes—an effect typically associated with composites modified using carbon fillers.

From a practical point of view, the optimal solution is a composite containing 7.5% carbonisate material and a resin–reinforcement ratio of 60/40, which ensures high hardness with acceptable impact strength and stable mechanical parameters.

The results obtained confirm that statistical analysis is an indispensable tool in the evaluation of epoxy composite properties, enabling quantitative determination of the influence of filler content and fraction on strength parameters and the determination of correlation relationships between hardness and impact strength.

The results are consistent with earlier studies [24], which showed that a 60/40 composite with 7.5% carbonisate (<500 µm) achieves the highest hardness and homogeneity. This confirms the effectiveness of carbonisate MDF pyrolysis as a filler that improves the stability and homogeneity of epoxy–glass composites.

The SEM examination further confirmed that higher carbonisate content improves interfacial bonding and laminate compactness, which explains the observed increase in hardness and the reduction in impact strength.

## Figures and Tables

**Table 1:** Characteristics of composites obtained by hand lamination technique.

No.	Layers of Mats	wt% Resin Ratio	wt% Content Glass Mats	wt% Carbonisate Ratio	Carbonisate Grain Size [μm]	Sample Designation
1	10	60	40	0	-	X
2	10	60	35	5	<500	X1
3	10	60	32.5	7.5	<500	X2
4	10	65	35	0	-	Y
5	10	65	30	5	<500	Y1
6	10	65	27.5	7.5	<500	Y2

**Table 2:** Mean impact strength values for composite samples.

Sample Designation	Impact Strength U, [kJ/m^2^]
X	51.23
X1	45.78
X2	43.56
Y	49.52
Y1	43.32
Y2	38.46

**Table 3:** Mean Barcol hardness values for composite samples.

Sample Designation	Barcol Hardness HBa
X	22.45
X1	31.43
X2	35.17
Y	35.97
Y1	36.25
Y2	30.84

**Table 4:** Calculated sample size (*n*) for impact and hardness tests.

Sample Designation	Number *n* (Impact)	Number *n* (Hardness)
Material 60/40 (X)	60	92
Material 60/40 (X1)	19	100
Material 60/40 (X2)	14	70
Material 65/35 (Y)	18	73
Material 65/35 (Y1)	18	51
Material 65/35 (Y2)	39	64

**Table 5:** Results of the Shapiro–Wilk normality test for the impact strength measurements.

Specimen ID	*p* Statistic Shapiro-Wilka
X	0.46006
X1	0.07384
X2	0.71502
Y	0.01238
Y1	0.20337
Y2	0.00088

**Table 6:** Normality assessment of hardness values using the Shapiro–Wilk test.

Specimen ID	*p* Statistic Shapiro-Wilka
X	0.00000
X1	0.00267
X2	0.00001
Y	0.00000
Y1	0.00000
Y2	0.00000

**Table 7:** *p*-values of the Kruskal–Wallis ANOVA test for impact strength measurements.

Sample Designation	Independent Variable (Grouping) Impact Strength; *p*-Value for Multiple Comparisons (Two-Tailed); Kruskala-Wallisa Test: H (5, N = 360) = 207.7443 *p* = 0.000					
	X	X1	X2	Y	Y1	Y2
X		0.000041	0.000000	1.000000	0.000000	0.000000
X1	0.000041		0.102511	0.000397	0.045222	0.000000
X2	0.000000	0.102511		0.000000	1.000000	0.000155
Y	1.000000	0.000397	0.000000		0.000000	0.000000
Y1	0.000000	0.045222	1.000000	0.000000		0.000503
Y2	0.000000	0.000000	0.000155	0.000000	0.000503	

**Table 8:** *p*-values of the Kruskal–Wallis ANOVA test for hardness measurements.

Sample Designation	Independent Variable (Grouping) Hardness; *p*-Value for Multiple Comparisons (Two-Tailed); Kruskala-Wallisa Test: H (5, N = 600) = 440.8020 *p* = 0.000					
	X	X1	X2	Y	Y1	Y2
X		0.000000	0.000000	0.000000	0.000000	0.000000
X1	0.000000		0.000000	0.000000	0.000000	1.000000
X2	0.000000	0.000000		1.000000	0.140833	0.000000
Y	0.000000	0.000000	1.000000		1.000000	0.000000
Y1	0.000000	0.000000	0.140833	1.000000		0.000000
Y2	0.000000	1.000000	0.000000	0.000000	0.000000	

**Table 9:** Measures of impact strength variability (dispersion) of the tested samples.

Indicators	Sample Designation					
	X	X1	X2	Y	Y1	Y2
Mean	51.23	45.78	43.56	49.52	43.32	38.46
Median	52.16	46.04	43.25	50.01	43.23	37.39
Standard deviation	5.43	3.04	2.60	2.94	2.90	4.35
Variance	29.44	9.26	6.77	8.62	8.40	18.95
Minimum	35.25	39.83	36.63	38.78	37.72	31.27
Maximum	63.47	56.85	49.36	54.70	49.50	55.70
Q1 First quartile (25%)	48.00	43.38	41.86	47.85	40.90	35.49
Q3Third quartile (75%)	54.53	47.63	45.84	51.50	45.79	40.37
Vs	10.59	6.65	5.97	5.93	6.69	11.32
V_Q_	6.27	4.61	4.59	3.65	5.65	6.53

**Table 10:** Measures of hardness variability (dispersion) of the tested samples.

Indicators	Sample Designation					
	X	X1	X2	Y	Y1	Y2
Mean	22.45	31.43	35.19	35.97	36.25	30.84
Median	22.00	31.00	35.00	35.00	36.00	30.00
Standard deviation	2.46	2.58	2.15	2.19	1.83	2.05
Variance	6.05	6.67	4.64	4.82	3.36	4.20
Minimum	20.00	26.00	30.00	30.00	34.00	28.00
Maximum	32.00	38.00	40.00	40.00	40.00	35.00
Q1 First quartile (25%)	20.00	30.00	33.75	35.00	35.00	29.00
Q3Third quartile (75%)	24.00	33.00	37.00	38.00	38.00	33.00
Vs	10.95	8.22	6.12	6.10	5.06	6.64
V_Q_	9.09	4.84	4.64	4.29	4.17	6.67

## Data Availability

The raw data supporting the findings of this study are protected by a granted patent and cannot be made publicly available due to legal restrictions related to intellectual property rights. Data may be provided by the corresponding author upon reasonable request and subject to a confidentiality agreement.
